# Clinico-Biochemical Profile and Identification of Independent Risk Factors of Frequent Relapse in Childhood-Onset Steroid-Sensitive Nephrotic Syndrome

**DOI:** 10.7759/cureus.21765

**Published:** 2022-01-31

**Authors:** Manas Ranjan Behera, Ch. Manoj Kumar, Seba Ranjan Biswal, P. Vinod K Reddy, Gundreddy Bala Praneeth Reddy, Nikhitha Polakampalli, Ravi Kumar, Sanjay Kumar Sahu

**Affiliations:** 1 Pediatrics, Kalinga Institute of Medical Sciences, Bhubaneswar, IND

**Keywords:** urinary protein-to-creatinine ratio, frequent relapse, steroid sensitive, serum albumin, relapse, edema, nephrotic syndrome

## Abstract

Background and aim

Nephrotic syndrome is one of the commonest glomerular diseases in children, and the majority of them have minimal change lesions in histology with a favorable outcome. Most children with minimal change disease (MCD) are steroid-sensitive, but half of them have a frequent relapse and a prolonged course. This study was conducted to evaluate the clinical manifestations and biochemical profile and to determine independent risk factors of frequent relapse in children with steroid-sensitive nephrotic syndrome (SSNS).

Methods

This was a tertiary care hospital-based observational study conducted at the pediatric department of Kalinga Institute of Medical Sciences (KIMS), Bhubaneswar, India, from October 2017 to September 2019. Fifty-three children from age one to 15 years admitted with steroid-sensitive nephrotic syndrome diagnosed as per the International Study of Kidney Disease in Children (ISKDC) criteria were enrolled in the study. On admission, history-taking, physical examination, and routine hematological and biochemical tests were carried out. Children who had no infection were started oral prednisolone at the dose of 2 mg/kg/day for six weeks, followed by 1.5 mg/kg/day on alternate days for six weeks with daily follow-up for evidence of proteinuria till remission. The parameters evaluated were age at presentation, sex, type of presentation, precipitating factors, laboratory findings, and rapidity of steroid response. All children were followed up for one year, and those with no relapse over a period of one year after remission served as the control group to determine the risk factors for relapse. Data were analyzed using standard statistical software (Stata version 13.1, StataCorp LLC, College Station, Texas, USA).

Results

Of the 53 cases, 47% of the children had a relapse. In the relapse category, 88% were male, and 67% were between one and 5.5 years. The clinical manifestations during the first episode in the relapse group were similar to the no relapse group. Investigations revealed that 64% of the children with relapse had serum total protein ≤ 4.2 g/dL (p = 0) and that 59% had serum albumin ≤ 1.8 g/dL (p = 0.004). In the relapse group, 41% of the children went into remission within two weeks of initiation of therapy as compared with 80% in the no relapse group.

Conclusion

The risk factors determined for relapse in SSNS are male sex, younger age, low serum albumin, low serum total protein, and delayed response to steroid therapy.

## Introduction

Nephrotic syndrome is one of the frequently encountered glomerular diseases usually affecting children between two and six years. It manifests with heavy or nephrotic range proteinuria (>3.5 g/24 hours or >40 mg/m^2^/hour) or a spot urine protein-to-creatinine ratio of >2, along with the triad of hypoalbuminemia (serum albumin < 2.5 g/dL), edema, and hyperlipidemia (serum cholesterol > 200 mg/dL) [1]. About 80% of children with nephrotic syndrome have minimal change lesions in renal histology and have a favorable outcome. The majority of children with idiopathic nephrotic syndrome undergo remission following appropriate therapy with oral prednisolone, hence termed as steroid-sensitive nephrotic syndrome (SSNS) [2]. Most children with minimal change disease (MCD) are steroid-sensitive; however, 40%-50% show frequent relapse and a prolonged course. Relapse in steroid-sensitive nephrotic syndrome (SSNS) usually follows an upper respiratory tract or gastrointestinal infection, and in the developing countries, in 52%-70% of cases, it follows an upper respiratory tract infection [3]. Frequent relapses are associated with an increased risk of complications due to steroid therapy, including life-threatening infections. Despite the initial response rate of 90%-95% to corticosteroid, with a favorable prognosis, SSNS relapses in 60%-90% of initial responders, increasing morbidity and complications and decreasing the quality of life [4-6]. At the onset of the disease process, there is uncertainty regarding relapse and the course of the disease process. Predictors of relapse may lead to better management strategies and better short-term and long-term outcomes. Therefore, this study was conducted to assess the clinical spectrum and biochemical profile and to determine the independent risk factors of frequent relapse in SSNS in children.

## Materials and methods

This was an observational study conducted at the pediatric department of Kalinga Institute of Medical Sciences (KIMS), Bhubaneswar, India, from October 2017 to September 2019 after approval of the Institutional Ethics Committee of KIMS. The primary objective was to assess the clinical spectrum and biochemical profile of children with SSNS, and the secondary objective was to find out independent risk factors for frequent relapse in SSNS in children. Fifty-three children from one to 15 years admitted with steroid-sensitive nephrotic syndrome diagnosed as per the International Study of Kidney Disease in Children (ISKDC) criteria were enrolled in the study. Children with congenital nephrotic syndrome, secondary nephrotic syndrome, and steroid-resistant nephrotic syndrome, on immunosuppressive agents other than steroids, and with primary immunodeficiency diseases were not included in the study.

On admission, meticulous history-taking and a thorough physical examination were carried out for all children enrolled in the study. Routine hematological and biochemical tests including complete blood count, C-reactive protein, renal function tests, serum electrolytes, liver function tests, lipid profile, urine analysis, spot urine protein-to-creatinine ratio, chest X-ray, Mantoux test, and ultrasonography of the abdomen were done for all children. Blood and urine cultures were sent in those with evidence of infection. Antibiotics were started in children who had evidence of infection prior to the start of corticosteroid. Children who had no remission in spite of adequate infection control and who had no infection were started oral prednisolone at the dose of 2 mg/kg/day for six weeks, followed by 1.5 mg/kg/day on alternate days for six weeks. Follow-up was done every day by first morning voided urine sample for evidence of proteinuria till remission. The number of weeks for remission after starting of steroid was documented. The children were also monitored for improvement, deterioration, and development of complications.

On discharge, parents were counseled regarding the nature of the disease and symptoms of relapse and were advised for follow-up at KIMS, Bhubaneswar, during relapse for evaluation and to know the frequency of relapse in a period of one year since the first attack.

Data were collected in a standard case record proforma. The parameters evaluated were age at presentation, sex, type of presentation, precipitating factors, laboratory findings, and rapidity of steroid response.

Out of the 53 cases of SSNS, children with no relapse over a period of one year after remission served as the control group to determine the risk factors for relapse.

The data collected were then presented as measures of number or percentage. The Chi-square test or Fisher’s extract test was used wherever applicable for testing the significance of the statistical difference between parameters with the use of probability value P. Statistical significance was defined as p value < 0.05. Data were analyzed using standard statistical software (Stata version 13.1, StataCorp LLC, College Station, Texas, USA).

## Results

**Figure 1:**
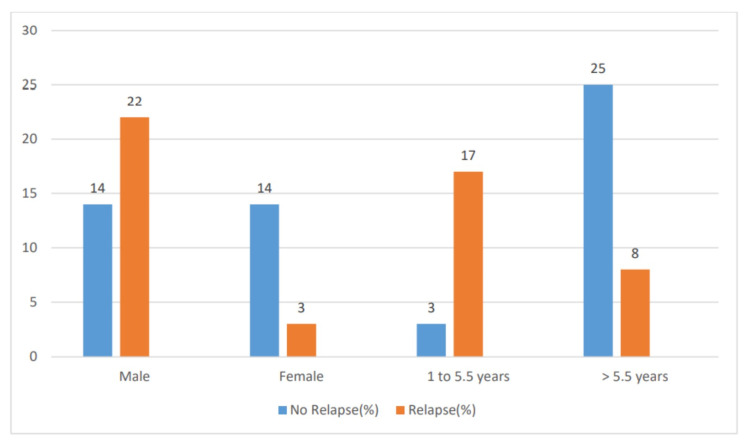
Age and sex distribution

The study was conducted from October 2017 to September 2019 over a period of two years. During the study period, there were 19,553 pediatric admissions, of which there were 61 cases of nephrotic syndrome. Among these, 53 cases were enrolled in the study as per the criteria for inclusion, and of the 53 cases, 36 (68%) were males and 17 (32%) were females. Relapse was seen in 25 (47%) cases, whereas 28 (53%) cases had no relapse. Of the 25 children who had a relapse, 22 (88%) were male and only three (12%) were females (p = 0.003), whereas in children with no relapse, 50% were males and 50% were females. The children were divided into five groups as per their age. Relapse was seen in 16 (64%) children in the age group of one to three years, five (20%) in three to six years, three (12%) in six to nine years, one (4%) in nine to 12 years, and none in >12 years. There was no relapse in three (11%) children in the age group of one to three years, 13 (46%) in three to six years, nine (32%) in six to nine years, two (7%) in nine to 12 years, and one (4%) in >12 years. Most of the children (16, 64%) in the age group of one to three years had a relapse, whereas most of the children (13, 46%) in the age group of three to six years had no relapse. The mean age at onset of illness in the relapse group was 3.8 years, whereas it was 6.5 years in the no relapse group (p = 0.001). Of the children who had a relapse, 67% were between one and 5.5 years of age, whereas 90% of the children above 5.5 years had no relapse (p = 0.001) (Table 1 and Figure 1).

**Table 1:** Age and sex distribution

Parameters	No relapse (%)	Relapse (%)	p
Male	14 (50%)	22 (88%)	0.003
Female	14 (50%)	3 (12%)	
1–5.5 years	3 (10%)	17 (67%)	<0.001
>5.5 years	25 (90%)	8 (33%)	

Almost half of the cases in the relapse group (56%) and no relapse group (46%) belong to the lower middle class strata.

**Figure 2:**
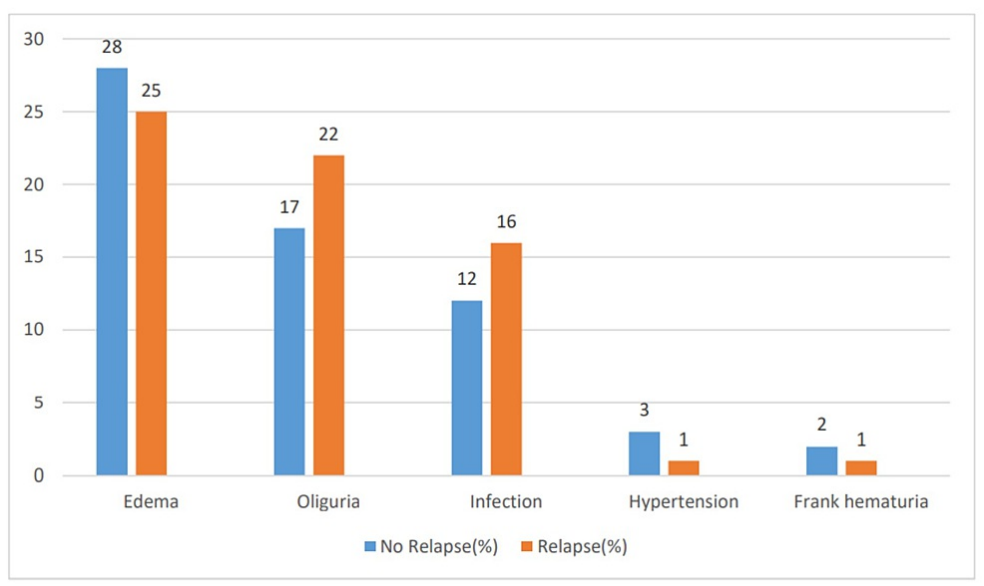
Clinical presentation in the first attack of steroid-sensitive nephrotic syndrome

The clinical manifestations during the first attack of SSNS in children with relapse were edema (25, 100%), oliguria (22, 88%), infection (16, 64%), hypertension (1, 4%), and hematuria (1, 4%). The children in the no relapse group presented with edema (28, 100%), oliguria (17, 61%), infection (12, 43%), hypertension (3, 10%), and hematuria (2, 7%) (Table 2 and Figure 2).

**Table 2:** Clinical presentation in the first attack of steroid-sensitive nephrotic syndrome

Clinical features	No relapse (%)	Relapse (%)	Total
Edema	28 (100%)	25 (100%)	53 (100%)
Oliguria	17 (61%)	22 (88%)	39 (74%)
Infection	12 (43%)	16 (64%)	28 (53%)
Hypertension	3 (10%)	1 (4%)	4 (8%)
Frank hematuria	2 (7%)	1 (4%)	3 (6%)

The incidence of infection during the first attack of SSNS in children with no relapse was 12 (42.8%), whereas it was 16 (66.6%) during the first relapse in children who had a relapse (p = 0.124). The focus of infection in the relapse group was the respiratory tract (63%), whereas it was predominantly genitourinary (83%) in the no relapse group. Hematuria was present in six (24%) children who had a relapse and four (14%) children with no relapse (p = 0.488).

Investigations sent during the first attack of SSNS revealed that 16 (64%) children who had a relapse had serum total protein ≤ 4.2 g/dL as compared with 22 (80%) children in the no relapse group who had serum total protein > 4.2 g/dL (p = 0). Similarly, 15 (59%) of the children with relapse had serum albumin ≤ 1.8 g/dL as compared with 22 (80%) children in the no relapse group who had serum albumin > 1.8 g/dL (p = 0.004).

**Figure 3:**
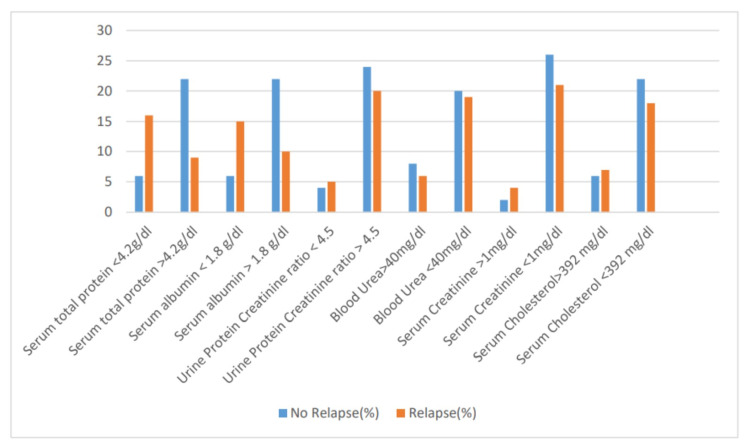
Biochemical parameters

Spot urinary protein-to-creatinine ratio > 4.5 was found in 20 (80%) children with relapse, whereas it was 24 (86%) in the no relapse group (p = 0.719). Blood urea > 40 mg/dL was found in six (11%) children with relapse, whereas it was eight (15%) in the no relapse group (p = 0.706). Serum creatinine > 1 mg/dL was found in four (16%) children with relapse, whereas it was two (7%) in the no relapse group (p = 0.404). Serum cholesterol > 392 mg/dL was found in seven (28%) children with relapse, whereas it was six (21%) in the no relapse group (p = 0.579) (Table 3 and Figure 3).

**Table 3:** Biochemical parameters

Parameters	No relapse (%)	Relapse (%)	Total	p
Serum total protein < 4.2 g/dL	6 (20%)	16 (64%)	22 (42%)	0
Serum total protein > 4.2 g/dL	22 (80%)	9 (36%)	31 (58%)	
Serum albumin < 1.8 g/dL	6 (20%)	15 (59%)	21 (40%)	0.004
Serum albumin > 1.8 g/dL	22 (80%)	10 (41%)	32 (60%)	
Urine protein-to-creatinine ratio < 4.5	4 (14%)	5 (20%)	9 (17%)	0.719
Urine protein-to-creatinine ratio > 4.5	24 (86%)	20 (80%)	44 (83%)	
Blood urea > 40 mg/dL	8 (15%)	6 (24%)	14 (26%)	0.706
Blood urea < 40 mg/dL	20 (85%)	19 (76%)	39 (73%)	
Serum creatinine > 1 mg/dL	2 (7%)	4 (16%)	6 (11%)	0.404
Serum creatinine < 1 mg/dL	26 (93%)	21 (84%)	47 (89%)	
Serum cholesterol > 392 mg/dL	6 (21%)	7 (28%)	13 (24%)	0.579
Serum cholesterol < 392 mg/dL	22 (79%)	18 (72%)	40 (76%)	

**Figure 4:**
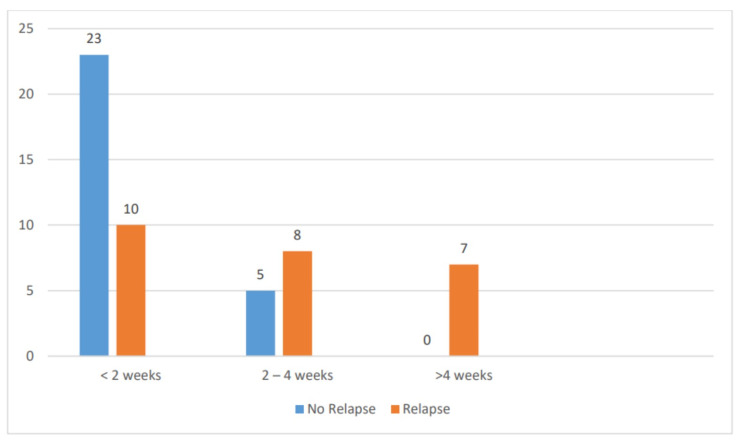
Duration of response to steroid

On treatment with oral prednisolone, 33 (62%) children went into remission within two weeks of initiation of therapy, 13 (24%) took two to four weeks, and seven (14%) took >4 weeks. In the relapse group, 10 (41%) children went into remission within two weeks of initiation of therapy, eight (33%) took two to four weeks, and seven (26%) took >4 weeks. In the group with no relapse, 23 (80%) children went into remission within two weeks of initiation of therapy and five (20%) took two to four weeks (p = 0.001). The mean duration for remission among the relapse cases was 11.48 days, whereas it was 10.14 days in children with no relapse (p = 0.478) (Table 4 and Figure 4).

**Table 4:** Duration of response to steroid

Weeks to remission	No relapse (%)	Relapse (%)	Total	p
<2 weeks	23 (80%)	10 (41%)	33 (62%)	0.001
2–4 weeks	5 (20%)	8 (33.4%)	13 (24%)	
>4 weeks	0	7 (25.6%)	7 (14%)	
Mean duration to remission	10.14 + 4.503	11.48 + 8.292		0.478

## Discussion

This was an observational study conducted at the Department of Pediatrics of Kalinga Institute of Medical Sciences, Bhubaneswar, Odisha, India, from October 2017 to September 2019. Out of 61 cases of nephrotic syndrome, 53 cases of SSNS were included in the study, and eight were excluded because three were steroid-resistant and five had a congenital nephrotic syndrome. Of the 53 cases, those who had no relapse for one year after the first remission served as the control group to determine the risk factors for relapse. Therefore, out of the 53 cases of SSNS, 28 (53%) had no relapse and 25 (47%) had a relapse. Mishra et al. had observed in their study that 40.7% of children had no relapse, whereas 59.3% had a relapse [7]. Frequent relapse was seen in one-fourth of the children who had a relapse in this study, similar to the ISKDC report, but in contrast to the findings of Constantinescu et al. (2000), who observed it to be around 60% [5,8].

In this study, two-thirds of the cases were boys, with a male-to-female ratio of 2.1:1, which is consistent with the 1978 ISKDC report and the findings of Constantinescu et al. (2000), which showed a male-to-female ratio of 2:1 and 1.8:1, respectively [8,9].

On analyzing the sex distribution among the relapse and no relapse group, in the no relapse group, boys and girls were equally distributed with 50% in each group. However, in the group with a relapse, the majority were boys (88%), and on analysis, it was statistically significant, indicating the male sex to be a predictor for relapse. Similar findings were also endorsed by Sureshkumar et al. and Andersen et al. [10,11].

Almost 75% of the children presented between one to six years of age, with the peak incidence being between the age of one and three years. The mean age at presentation among the relapse and no relapse groups were 3.83 and 6.57 years, respectively, which was found to be statistically significant (p = 0.001). The majority of the children with no relapse were above 5.5 years as compared with children who had a relapse, who were between one and 5.5 years, which was statistically significant (p < 0.001). This signifies that the age of onset of below 5.5 years is a significant predictor of relapse.

Takeda et al. (1996) found that the incidence of the first attack of SSNS was 49% in the age group of one to three years, 24% in four to six years, and 27% above seven years [12]. Noer (2005) had a similar finding of 60% belonging to one to six years and 40% above six years [13]. The findings of both studies are in concordance with the present study.

The high incidence of SSNS in children from one to six years can be attributed to the fact that 75% of MCD cases have their onset between two and six years and that MCD comprises 85% of the total cases of nephrotic syndrome in childhood [1,14].

On analysis of clinical manifestations, edema was the universal presentation seen in 100% of cases; three-quarters of them had oliguria, and half of them had some focus of infection. On comparing the relapse and no relapse groups, no statistical significance was observed, similar to the observations made by Srivastava et al. (1975) [15]. Only less than 10% of the children in this study presented with hypertension and hematuria; however, Noer (2005) reported hypertension in 22% of children, and Constantinescu et al. (2000) reported hematuria in 12.35% of children with SSNS [8,13].

Noer (2005) also observed that the incidence of infection in the first relapse was double as compared with the first attack, whereas there was no such difference in this study. It may be attributed to the use of over-the-counter antibiotics during the first relapse, which is a very common phenomenon in India. Genitourinary infections were the commonest infection, followed by respiratory infections, similar to the observations made by other studies [16,17].

The comparison of biochemical parameters serum total protein and serum albumin in the relapse and no relapse groups was statistically significant. A serum total protein level < 4.2 g/dL and serum albumin level < 1.8 g/dL were found to be significant predictors of relapse, similar to the observation of Takeda et al. [12].

On comparing the levels of spot urinary protein-to-creatinine ratio, blood urea, serum creatinine, and serum cholesterol in the two groups, no statistical significance was determined. Hence, these parameters do not serve as predictors for relapse.

The mean duration for remission was 10.14 days in children with no relapse, and 80% of the children went into remission within two weeks of initiation of therapy. Constantinescu et al. reported it to be 13 days and endorsed that those who responded within one week had a lesser probability of relapse [8].

In this study, the risk factors determined for relapse in SSNS include male sex, younger age of presentation, low level of serum albumin and serum total protein during the initial presentation, and finally delayed response to steroid therapy. Infections were the most common precipitating factors for relapse. These risk factors, if present during the first attack, will help identify the group of children who will relapse in the future; therefore, caregivers can be counseled, and better management strategies can be planned to minimize the chronicity of the disease and prevent end-stage renal disease. The major drawback of this study is it does not reflect the whole community as it was carried out in a single center with a limited number of population. Larger multicentric studies are required to make the result generalized.

## Conclusions

The risk factors determined for relapse in SSNS in this study include male sex, younger age of presentation, low level of serum albumin and serum total protein during the initial presentation, and finally delayed response to steroid therapy. Relapse in nephrotic syndrome increases morbidity and mortality and decreases the quality of life of the patient, intimidating caregivers and pediatricians. More multicentric studies with larger sample sizes can determine other predictors for relapse that can alter the course of the disease, and therapeutic trials can be planned with longer low-dose steroid regimens and steroid-sparing agents that can prevent relapse.
